# Angiogenesis extent and macrophage density increase simultaneously with pathological progression in B-cell non-Hodgkin's lymphomas

**DOI:** 10.1038/sj.bjc.6690154

**Published:** 1999-02

**Authors:** A Vacca, D Ribatti, L Ruco, F Giacchetta, B Nico, F Quondamatteo, R Ria, M Iurlaro, F Dammacco

**Affiliations:** Department of Biomedical Sciences and Human Oncology, University of Bari Medical School, Piazza Giulio Cesare 11, Bari, I-70124, Italy; Institute of Human Anatomy, Histology and Embryology, University of Bari Medical School, Piazza Giulio Cesare 11, Bari, I-70124, Italy; Department of Experimental Medicine and Pathology, University ‘La Sapienza’, Rome, I-00185, Italy; Consortium Cancer Research Center (CARSO), Valenzano-Bari, I-70126, Italy; Institute of Anatomy and Histology, Georg-August University, Göttingen, Germany

**Keywords:** angiogenesis, macrophage, non-Hodgkin's lymphoma, tumour progression

## Abstract

Node biopsies of 30 benign lymphadenopathies and 71 B-cell non-Hodgkin's lymphomas (B-NHLs) were investigated for microvessel and macrophage counts using immunohistochemistry and morphometric analysis. Both counts were significantly higher in B-NHL. Moreover, when these were grouped into low-grade and high-grade lymphomas, according to the Kiel classification and Working Formulation (WF), statistically significant higher counts were found in the high-grade tumours. Immunohistochemistry and electron microscopy revealed a close spatial association between microvessels and macrophages. Overall, the results suggest that, in analogy to what has already been shown in solid tumours, angiogenesis occurring in B-NHLs increases with tumour progression, and that macrophages promote the induction of angiogenesis via the release of their angiogenic factors. *© 1999 Cancer Research Campaign*


*British Journal of Cancer* (1999) **79**(5/6), 965–970
© 1999 Cancer Research Campaign
Article no. bjoc.1998.0154

### Angiogenesis extent and macrophage density increase simultaneously with pathological progression in B-cell non-Hodgkin's lymphomas

**A Vacca[1], D Ribatti[2], L Ruco[3], F Giacchetta[4], B Nico[2], F Quondamatteo[5], R Ria[1], M Iurlaro[1] and F Dammacco[1]**

[1]Department of Biomedical Sciences and Human Oncology, and [2]Institute of Human Anatomy, Histology and Embryology, University of Bari Medical School, Piazza Giulio Cesare 11, I-70124, Bari, Italy; [3]Department of Experimental Medicine and Pathology, University 'La Sapienza', I-00185, Rome, Italy; [4]Consortium Cancer Research Center (CARSO), I-70126, Valenzano-Bari, Italy; [5]Institute of Anatomy and Histology, Georg-August University, Göttingen, Germany

**Summary** Node biopsies of 30 benign lymphadenopathies and 71 B-cell non-Hodgkin's lymphomas (B-NHLs) were investigated for microvessel and macrophage counts using immunohistochemistry and morphometric analysis. Both counts were significantly higher in B-NHL. Moreover, when these were grouped into low-grade and high-grade lymphomas, according to the Kiel classification and Working Formulation (WF), statistically significant higher counts were found in the high-grade tumours. Immunohistochemistry and electron microscopy revealed a close spatial association between microvessels and macrophages. Overall, the results suggest that, in analogy to what has already been shown in solid tumours, angiogenesis occurring in B-NHLs increases with tumour progression, and that macrophages promote the induction of angiogenesis via the release of their angiogenic factors.

**Keywords:** angiogenesis; macrophage; non-Hodgkin's lymphoma; tumour progression

Progression (growth, invasion and metastasis) of solid tumours requires, and is correlated with, the formation of microvessels, i.e. angiogenesis (Folkman, 1995). Microvessels promote growth because they convey nutrients and oxygen and remove catabolites, while endothelial cells secrete paracrine growth factors for tumour cells (Hamada et al, 1992). Microvessels facilitate invasion because endothelial cells (at their tips) secrete several extracellular matrix-degrading enzymes (Mignatti and Rifkin, 1993). They also enable metastasis formation because an expanding endothelial surface offers tumour cells more opportunities to enter the circulation (Aznavoorian et al, 1993). Host inflammatory cells, including macrophages, have also been shown to be closely associated with progression because they stimulate both growth and angiogenesis via paracrine mechanisms, and facilitate invasion by secreting matrix-degrading enzymes (Hildenbrand et al, 1995; Polverini, 1996).

There is only fragmentary evidence of the extent to which the position is the same in haematological tumours. Angiogenesis is correlated with tumour growth (S-phase fraction) in multiple myelomas (Vacca et al, 1994) and with progression stages of mycosis fungoides (Vacca et al, 1997), and it is readily observed in B-cell and T-cell acute lymphoblastic leukaemia at diagnosis, but not after treatment (Perez-Atayde et al, 1997).

This paper presents the results of an investigation on angiogenesis extent and macrophage density in benign lymphadenophathies and in non-Hodgkin's lymphomas of B-cell lineage (B-NHL)s grouped according to well-defined pathways of progression.

*Received 25 March 1998*
*Revised 4 June 1998*
*Accepted 13 July 1998*

*Correspondence to:* A Vacca, Department of Biomedical Sciences and Human Oncology, Section of Internal Medicine and Clinical Oncology, Policlinico, Piazza Giulio Cesare 11, I-70124 Bari, Italy

### MATERIALS AND METHODS

### Tissues

Representative node samples of 71 B-NHLs and of 30 benign lymphadenopathies were studied (Table 1). Approval was obtained from the local ethics committee and with informed consent from patients prior to therapy.

The reactive lymphoid hyperplasias displayed either follicular hyperplasia (human immunodeficiency virus lymphadenitis, rheumatoid lymphadenopathies), paracortical hyperplasia (dermatopathic lymphadenopathies), or mixed hyperplasia (Epstein–Barr virus, cytomegalovirus and *Toxoplasma gondii* lymphadenitides); the atypical forms displayed follicular hyperplasia.

Samples were formalin fixed and paraffin embedded immediately after surgical removal and sectioned for histopathology, immunohistochemistry and electron microscopy.

### Antibodies and immunohistochemistry

A three-layer biotin–avidin–alkaline phosphatase system was used as described previously (Vacca et al, 1997). Briefly, 8-μm sections were deparaffinized by the xylene–ethanol sequence, treated with 0.1% trypsin (Sigma Chemical Co, St Louis, MO, USA) and sequentially incubated with: (1) primary antibodies, i.e. murine monoclonal antibodies (MAbs) against the endothelial cell marker factor VIII (MAb M616, Dako, Glostrup, Denmark), the monocyte–macrophage marker CD68 (MAb KP1, Dako), or against several markers of T-cells (CD2, CD3, CD4, CD7 and CD8) and B-cells (κ and λ Ig chains, CD19, CD20, CD22 and HLA-DR, all from Becton-Dickinson, Sunnyvale, CA, USA) for lineage typing of NHLs as described previously (Vacca et al, 1996); in negative control sections, the MAbs were replaced by an unrelated monoclonal IgG1 produced by the murine myeloma P3X63Ag8 (Vacca et al, 1997); (2) a secondary antibody, i.e. biotin-labelled horse

**Table 1** Clinical and histological characteristics of the patients

| | | Kiel grade | WF grade |
|---|---|---|---|
| *B-cell lymphomas (REAL classification)*[a] | 71 | | |
| Small lymphocytic | 12 | Kiel low-grade[d] ($n$ = 42) | WF low-grade[d] ($n$ = 27) |
| Average age (males/females) | 54 (8/4) | | |
| Stage I–II/III–IV (A/B status)[b] | 3/9 (9/3) | | |
| Follicle cell lymphoma | 25 | | |
| Average age (males/females) | 58 (18/7) | | |
| Stage I–II/III–IV (A/B status) | 9/16 (16/9) | | |
| Follicular grade I | 6 | | |
| Follicular grade II | 9 | | |
| Follicular grade III | 10 | | |
| Mantle cell lymphoma | 5 | | WF intermediate-grade ($n$ = 20) |
| Average age (males/females) | 61 (3/2) | | |
| Stage I–II/III–IV (A/B status) | 1/4 (3/2) | | |
| Diffuse large B-cell lymphoma | 18 | Kiel high-grade ($n$ = 29) | |
| Average age (males/females) | 60 (8/10) | | |
| Stage I–II/III–IV (A/B status) | 10/8 (11/7) | | |
| Precursor B-lymphoblastic leukaemia–lymphoma | 6 | | WF high-grade ($n$ = 24) |
| Average age (males/females) | 51 (2/4) | | |
| Stage I–II/III–IV (A/B status) | 3/3 (2/4) | | |
| Burkitt's lymphoma | 5 | | |
| Average age (males/females) | 50 (2/3) | | |
| Stage I–II/III–IV (A/B status) | 3/2 (3/2) | | |
| *Lymphadenopathies* | 30 | | |
| Average age (males/females) | 60 (16/14) | | |
| Reactive lymphoid hyperplasias | 16 | | |
| Lymphadenitides[c] | 8 | | |
| Dermatopathic lymphadenopathies | 4 | | |
| Rheumatoid lymphadenopathies | 4 | | |
| Atypical lymphoid hyperplasias | 14 | | |
| Associated with systemic lupus erythematosus | 9 | | |
| Associated with common variable immunodeficiency | 5 | | |

[a]REAL, Revised European–American Lymphoma classification (Chan, 1994). [b]According to the Ann Arbor system (Carbone et al, 1971). [c]Four caused by Epstein–Barr virus, two by human immunodeficiency virus, one by cytomegalovirus, three by *Toxoplasma gondii*. [d]Malignancy grades according to the Kiel Classification (Lennert and Feller, 1992) and the Working Formulation (WF) for Clinical Usage (1982). Five and 13 samples of diffuse large B-cell lymphomas were allocated to the WF intermediate-grade and high-grade respectively.

anti-mouse Ig (Vector, Burlingame, CA, USA); (3) streptavidin–alkaline phosphatase conjugate (Promega, Madison, WI, USA). After the sections were exposed to Fast blue chromogenic solution (Promega) and brown staining was developed, they were washed, weakly counterstained with Gill's haematoxylin No. 2 (Polysciences, Warrington, PE, USA), dehydrated with the ethanol sequence, mounted in Eukitt, coded and examined under a double-headed photomicroscope (Leitz Dialux 20, Leitz, Wetzlar, Germany) by two investigators.

### Microvessel and macrophage counts

Four to six 200× fields covering the whole of each of three non-adjacent sections (every fourth section within 12 serial sections) per sample were examined with a 144-intersection point square reticulum (0.78 $mm^2$) inserted in the eyepiece.

Care was taken to identify microvessels (capillaries and small venules) as transversally sectioned tubes with a single layer of endothelial cells, either without or with a lumen (not exceeding 10 μm), and either without or with a thin basement membrane. Each assessment was agreed upon in turn. These stained microvessels, useful markers of angiogenesis (Folkman et al, 1989), were counted with a planimetric point-count method (Elias and Hyde, 1983) modified to restrict counting to transversally cut microvessels occupying the reticulum intersection points (Ribatti et al, 1998). As their diameter was smaller than the distance between adjacent points, only one such microvessel could occupy a given point. It was thus sufficiently certain that a given microvessel was counted only once, even if several of its section planes were present. The method (Elias and Hyde, 1983) also makes allowances for the heterogeneous distribution of microvessels in tissues. Indeed, in line with other (Kittas et al, 1985) and our own observations (Vacca et al, 1996), lymphadenopathies display very few microvessels in follicles. These mainly surround the mantle zone, and are scattered throughout the paracortical area and cords of lymphocytes. In follicular low- and intermediate-grade B-NHLs, microvessels are also very rare in follicles, but they are numerous in uninvolved tissue both between and in areas representing either diffuse infiltration or tissue shown as uninvolved by immunohistochemistry. Low-grade small lymphocytic, diffuse intermediate-grade, and high-grade B-NHLs display microvessels irregularly scattered throughout the tumour tissue. As the whole of each of three non-adjacent sections

 © Cancer Research Campaign 1999

was analysed, and because the transversally sectioned microvessels hit the intersection points randomly, the modified method produced objective counts. Means ±1 standard deviation (s.d.) were determined for each section, sample and groups of samples.

Macrophages highlighted by CD68 staining were counted on 6–8 250× fields (0.25 $mm^2$) covering the whole of each of the three sections adjacent to those stained for microvessels, and calculated as means ±1 s.d. for each section, sample and groups of samples.

### Electron microscopy

Small pieces (~ 1 $mm^3$) of tissue were fixed in 3% glutaraldehyde in 0.1M phosphate-buffered saline (PBS) for 3 h, washed in the same buffer for 12 h, post-fixed in 1% osmium tetroxide, dehydrated in graded ethanols and embedded in Epon 812. Ultrathin sections were cut with a diamond knife on a LKB ultratome, stained with uranyl acetate followed by lead citrate and examined in a 9A Zeiss electron microscope (Zeiss, Oberkochen, Germany).

### Statistics

The significance of changes in microvessel and macrophage counts in each group was assessed by parametric (Fisher's test) and non-parametric (Kruskal–Wallis' test) analysis of variance. Because this showed that some changes were significant, it was followed by Duncan's, Bonferroni's and Wilcoxon's ($t$) tests (at 95% confidence interval) to compare groups two by two. Correlations between microvessel and macrophage counts were assessed with the Pearson's ($r$) coefficient and simple regression analysis. Mean ± 1 s.d., medians and intervals of variation were also determined. Data were computed with Statistical Analysis Software (SAS, SAS Institute, Cary, NC, USA).

### RESULTS

Table 2 shows the counts of microvessels and macrophages on adjacent tissue sections of benign lymphadenopathies and B-NHLs as a whole and grouped in the Kiel and WF malignancy grades. The comparison of microvessel counts between groups revealed statistically significant differences (chi-squared = 46.3, d.f. = 3, $P < 0.001$, $F = 62.9$, $P < 0.001$). When differences were sought between groups, significantly higher counts were found in overall B-NHLs compared with lymphadenopathies ($P < 0.001$). Assessment by grades showed a significant increase in Kiel low-grade tumours compared with lymphadenopathies ($P < 0.01$) and yet another in high-grade tumours ($P < 0.01$). Similar increments were found in WF low-grade tumours over lymphadenopathies and in intermediate-grade over low-grade tumours, and (albeit not significant) in high-grade tumours. In parallel, the macrophage counts varied significantly between groups (chi-squared = 44.7, d.f. = 3, $P < 0.001$; $F = 69.1$, $P < 0.001$). The intergroup comparisons showed that the counts increased significantly in B-NHLs as a whole compared with lymphadenopathies ($P < 0.001$). Also, they were significantly higher in Kiel low-grade B-NHLs than in lymphadenopathies ($P < 0.05$), and in high-grade B-NHLs than in low-grade B-NHLs ($P < 0.01$). Similar relationships were found to the WF malignancy grades. The differences in microvessel and macrophage density between representative tissue samples are also shown in Figure 1. The within-group comparisons showed that both counts were always significantly correlated (Figure 2).

Macrophages were generally scattered throughout the B-NHL tissue, closely interwoven with tumour cells, and many rested in the interstitial stroma near or around blood or lymphatic capillaries (Figure 3A). There was a very close relationship between macrophages and microvascular endothelial cells, as assessed by electron microscopy (Figure 3B).

### DISCUSSION

The results show that B-NHLs have a higher degree of vascularization than benign lymphadenopathies, and that high-grade lymphomas have a higher vessel density than those of low grade. The results also show that macrophage infiltration increases in parallel with malignancy grade and is highly correlated with the extent of angiogenesis.

The association between angiogenesis and Kiel and WF malignancy grades suggests that angiogenesis increases with tumour progression in B-NHLs. These grading systems map out a progression pathway marked by large increments in tumour cell growth, as evaluated by S-phase fraction. Kiel low-grade B-NHLs display S-phase fractions <5%, whereas Kiel high-grade tumours (Czader et al, 1995; Mochen et al, 1997) and those in transition from WF low- to intermediate- and high-grade exhibit S-phase fractions ≥ 10% (Joensuu et al, 1990). Because angiogenesis favours tumour growth (Fox, 1997), this could thus complete a positive loop in B-NHLs.

B-NHL-associated angiogenesis requires endothelial cell proliferation and chemotaxis (needed for vascular sprouting) (Risau, 1997), probably induced by secretion of angiogenic factors (Watanabe et al, 1997). Both haematic and lymphatic endothelial cells of node tissues have been shown to proliferate and migrate in vitro in response to angiogenic factors such as interleukin 6 (IL-6), basic fibroblast growth factor (FGF-2) and vascular endothelial growth factor (VEGF-A) (Pepper et al, 1994). B-NHL cells have been shown to secrete IL-6 (Brach and Hermann, 1992), IL-8 (di Celle et al, 1994), FGF-2, VEGF-A (Vacca et al, 1998) and tumour necrosis factor-α (TNF-α) (Adami et al, 1994). In addition, the peritumoral inflammatory infiltrate surrounding the newly formed small blood and lymphatic vessels in the stroma of B-NHLs consists of fibroblasts, macrophages and other leukocytes that may contribute to the induction of the angiogenic response by secreting these and other factors (Folkman and Brem, 1992). Macrophages are strikingly associated with angiogenesis, as found in chronic inflammation associated with rheumatoid arthritis (Koch et al, 1986), psoriasis (Wolfe, 1989), atherosclerotic plaque (Sueishi et al, 1990) and in tumours, namely colon (Takahashi et al, 1996), breast (Leek et al, 1996), ovarian (Sheid, 1992) and renal (Xu et al, 1997) carcinomas. In tumours, macrophages are recruited and activated via several factors secreted by tumour cells: the chemokines (Rollins, 1997), as well as FGF-2 and VEGF-A (Watanabe et al, 1997), which are operative at picomolar concentrations. In both inflammatory and tumour tissues, activated macrophages have been shown to secrete several angiogenic factors, such as IL-8, FGF-2, VEGF-A, TNF-α, epidermal growth factor, tissue factor, hepatocyte growth factor/scatter factor and insulin-like growth factor-1 (Sunderkötter et al, 1994; Leek et al, 1997). Tentatively, we suggest that an increasing number of macrophages may be recruited and activated locally by more malignant B-cells, and that angiogenesis associated with B-NHLs may be induced, at least partly, by macrophages via their angiogenic factors.

© Cancer Research Campaign 1999

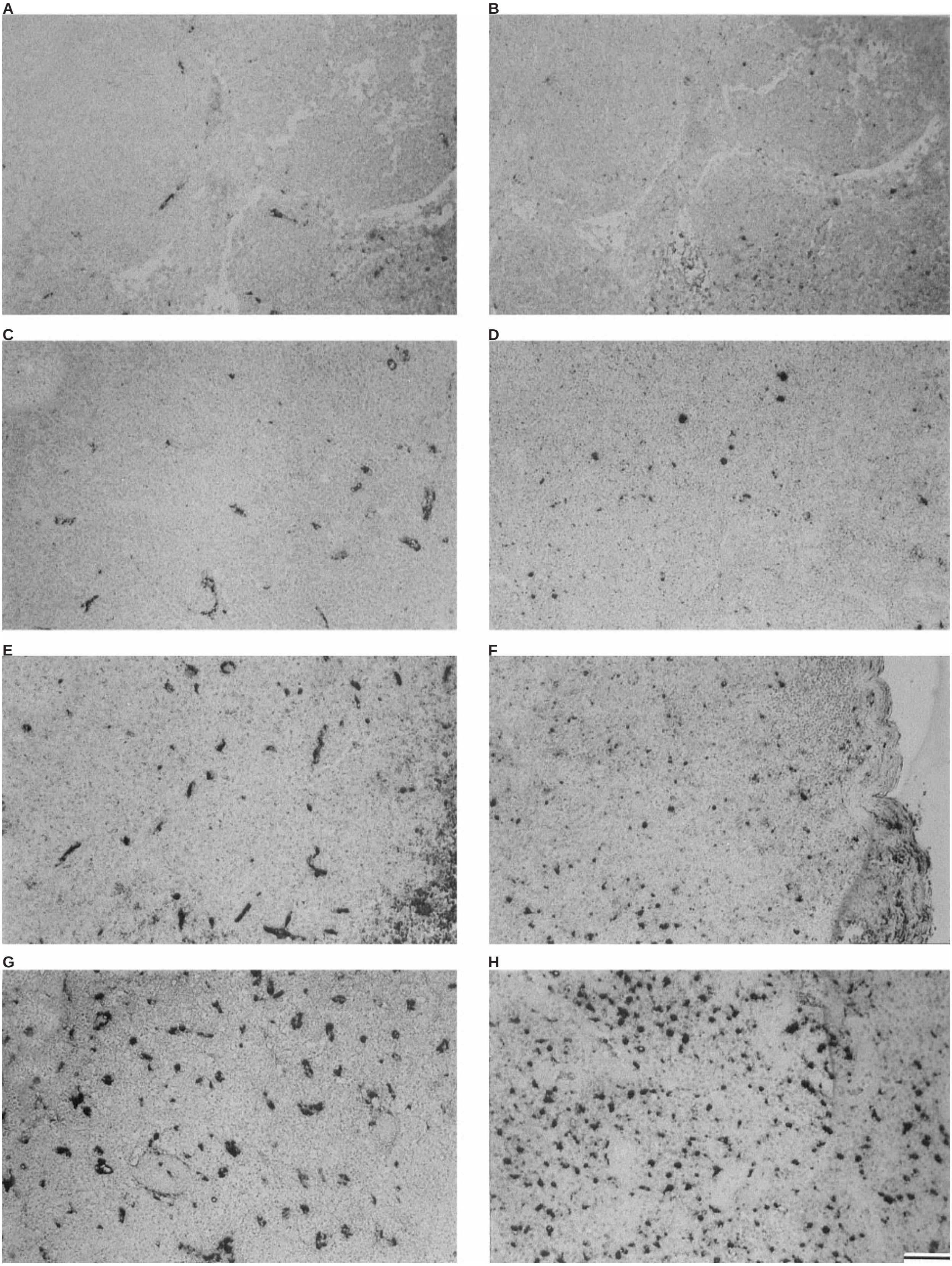

**Figure 1** Adjacent sections of a benign lymphadenopathy and B-NHL nodes stained with factor VIII for microvessels (**A**, **C**, **E**, **G**) and with CD68 for macrophages (**B**, **D**, **F** and **H**). (**A** and **B**) Reactive lymphoid hyperplasia. (**C** and **D**) WF low-grade (REAL, small lymphocytic). (**E** and **F**) WF intermediate-grade (REAL, mantle cell lymphoma). (**G** and **H**) WF high-grade (REAL, diffuse large B-cell lymphoma) B-NHL. Note the progressive increase of microvessels and macrophages from **A** and **B** to **G** and **H**. Bar = 30 μm

 *© Cancer Research Campaign 1999*

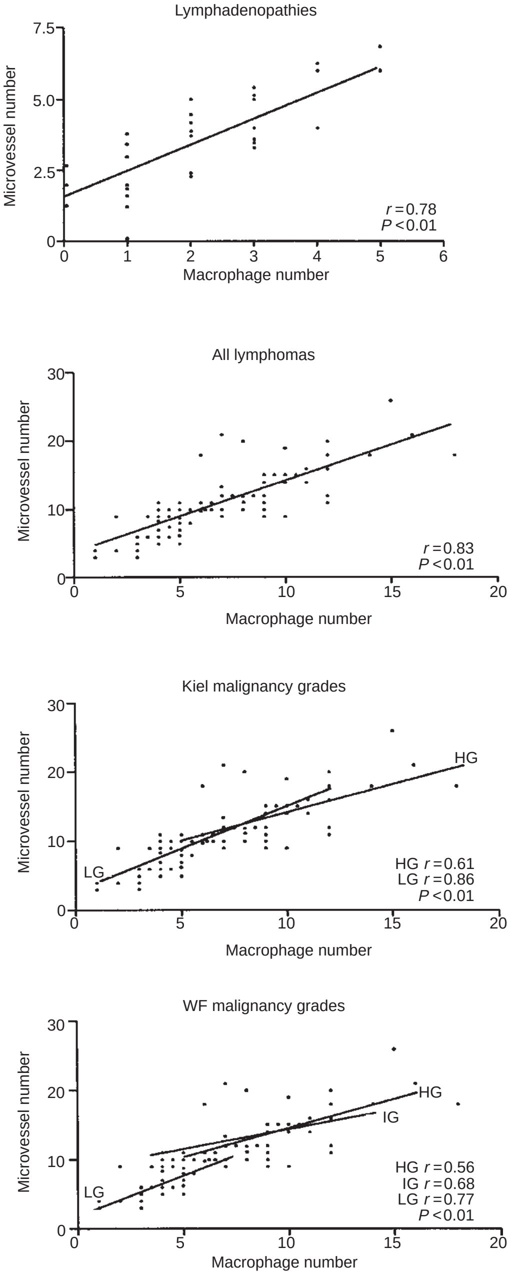

**Figure 2** Comparison of microvessel and macrophage counts. Significance of the regression analysis was calculated by Pearson's *r*-test. LG, low-grade; IG, intermediate-grade; HG, high-grade

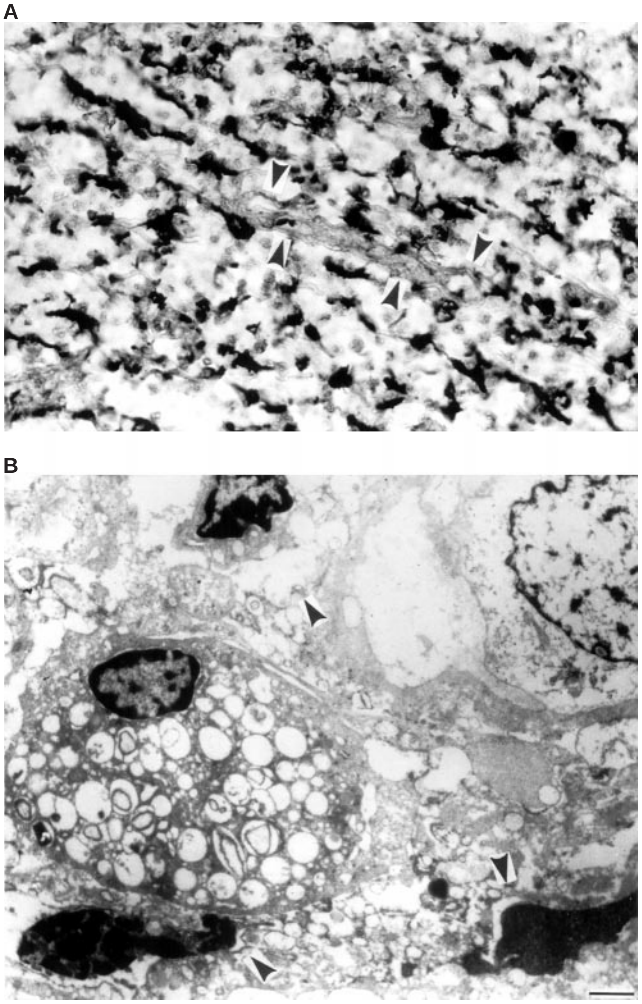

**Figure 3** Sections of a high-grade (REAL, diffuse large B-cell lymphoma) B-NHL node showing (**A**) numerous CD68-reactive macrophages irregularly scattered throughout the tumour and in close association with a microvessel (arrowhead), and (**B**) a macrophage in close vicinity with microvessels (arrowhead) by electron microscopy. Bar in **A** = 6 μm, in **B** = 0.07 μm

**Table 2** Tissue density of microvessels and macrophages

| | Benign lymphadenopathies | B-cell non-Hodgkin's lymphomas | | | | | |
|---|---|---|---|---|---|---|---|
| | | All | Kiel | | Working formulation | | |
| | | | Low-grade | High-grade | Low-grade | Intermediate-grade | High-grade |
| **No. of** | ***n* = 30** | ***n* = 71** | **(*n* = 42)** | **(*n* = 29)** | **(*n* = 27)** | **(*n* = 20)** | **(*n* = 24)** |
| Microvessels | 3 ± 1 | 11 ± 5*** | 8 ± 3** | 14 ± 4† | 7 ± 2** | 12 ± 3† | 14 ± 5 |
| (per 0.78 mm²) | (4, 1–6) | (10, 3–26) | (9, 3–18) | (14, 8–26) | (7, 3–11) | (12, 9–18) | (14, 8–26) |
| Macrophages | 2 ± 1 | 6 ± 3*** | 5 ± 2* | 9 ± 3† | 4 ± 1* | 7 ± 3† | 10 ± 3† |
| (per 0.25 mm²) | (2, 0–5) | (6, 1–18) | (4, 1–12) | (9, 5–18) | (4, 1–7) | (7, 3–14) | (10, 5–18) |

Results are expressed as mean ± 1 s.d. (median, interval of variation). ***$P < 0.001$, **$P < 0.01$ and *$P < 0.05$ compared with benign lymphadenopathies; †$P < 0.01$ compared with the preceding group.

© Cancer Research Campaign 1999

### ACKNOWLEDGEMENTS

This study was supported in part by the Associazione Italiana per la Ricerca sul Cancro (AIRC, Project Diagnosis and Prognosis in Clinical Oncology), Milan, Italy, and by the Ministry of Education, Rome, Italy.

### REFERENCES

Adami F, Guarini A, Pini M, Siviero F, Sancetta R, Massaia M, Trentin L, Foà R and Semenzato G (1994) Serum levels of tumour necrosis factor-α in patients with B-cell chronic lymphocytic leukemia. *Eur J Cancer* **30**A: 1259–1263

Aznavoorian S, Murphy AN, Stetler-Stevenson WG and Liotta LA (1993) Molecular aspects of tumor cell invasion and metastasis. *Cancer* **71**: 1368–1383

Brach MA and Hermann F (1992) Interleukin 6: present and future. *Int J Clin Lab Res* **22**: 143–151

Carbone PP, Kaplan HP, Mushof K, Smithers DW and Tubiana M (1971) Report of the Committee on Hodgkin's disease staging classification. *Cancer Res* **31**: 1860–1861

Chan JKC (1994) A new classification of lymphomas: the Revised European–American Lymphoma classification. *Adv Anat Pathol* **3**: 166–172

Czader M, Porwit A, Tani E, Ost A, Mazur J and Auer G (1995) DNA image cytometry and the expression of proliferative markers (proliferating cell nuclear antigen and Ki67) in non-Hodgkin's lymphomas. *Mod Pathol* **8**: 51–58

di Celle FP, Carbone A, Marchis D, Zhou D, Sozzani S, Zupo S, Pini M, Mantovani A and Foà R (1994) Cytokine gene expression in B-cell chronic lymphocytic leukemia: evidence of constitutive interleukin-8 (IL-8) mRNA expression and secretion of biologically active IL-8 protein. *Blood* **84**: 220–228

Elias H and Hyde DM (1983) Stereological measurement of isotropic structures. In *A Guide to Practical Stereology*, Elias H and Hyde DM (eds), pp. 25–43. Karger: Basel

Folkman J (1995) Angiogenesis in cancer, vascular, rheumatoid and other disease. *Nature Med* **1**: 27–31

Folkman J and Brem H (1992) Angiogenesis and inflammation. In *Inflammation: Basic Principles and Clinical Applications*, Gallin J, Goldstein M and Snyderman R (eds), pp. 821–839. Raven Press: New York

Folkman J, Watson K, Ingber D and Hanahan D (1989) Induction of angiogenesis during the transition from hyperplasia to neoplasia. *Nature* **339:** 58–61

Fox SB (1997) Tumour angiogenesis and prognosis. *Histopathology* **30**: 294–301

Hamada J, Cavanaugh PG and Lotan O (1992) Separable growth and migration factors for large-cell lymphoma cells secreted by microvascular endothelial cells derived from target organs for metastasis. **66**: 349–354

Hildenbrand R, Dilger I, Horlin A and Stutte HJ (1995) Urokinase and macrophages in tumour angiogenesis. *Br J Cancer* **72**: 818–823

Joensuu H, Klemi PJ and Jalkanen S (1990) Biologic progression in non-Hodgkin's lymphoma. A flow cytometric study. *Cancer* **65**: 2564–2571

Kittas C, Hansmann ML, Borisch B, Feller AC and Lennert K (1985) The blood microvasculature in T-cell lymphomas. A morphological, ultrastructural and immunohistochemical study. *Virchows Arch (Pathol Anat)* **405**: 439–452

Koch AE, Polverini PJG and Leibovich SJ (1986) Stimulation of neovascularization by human rheumatoid synovial tissue macrophages. *Arthritis Rheum* **29**: 471–479

Leek RD, Lewis CE, Whitehouse R, Greenall M, Clarke J and Harris AL (1996) Association of macrophage infiltration with angiogenesis and prognosis in invasive breast carcinoma. *Cancer Res* **56**: 4625–4629

Leek RD, Lewis CE and Harris AL (1997) The role of macrophages in tumour angiogenesis. In *Tumour Angiogenesis*, Bicknell R, Lewis CE and Ferrara N (eds), pp. 81–89. Oxford University Press: Oxford

Lennert K and Feller AC (1992) *Histopathology of Non-Hodgkin's Lymphomas*. (based on the Updated Kiel Classification). Springer: Berlin

Mignatti P and Rifkin DB (1993) Biology and biochemistry of proteinases in tumor invasion. *Physiol Rev* **73**: 161–195

Mochen C, Giardini R, Costa A and Silvestrini R (1997) MIB-1 and S-phase cell fraction predict survival in non-Hodgkin's lymphomas. *Cell Prolif* **30**: 37–47

Pepper MS, Wasi S, Ferrara N, Orci L and Montesano R (1994) In vitro angiogenic and proteolytic properties of bovine lymphatic endothelial cells. *Exp Cell Res* **210**: 298–305

Perez-Atayde AR, Sallan SE, Tedrow U, Connors S and Folkman J (1997) Spectrum of tumor angiogenesis in bone marrow of children with acute lymphoblastic leukemia. *Am J Pathol* **150**: 815–821

Polverini PF (1996) How the extracellular matrix and macrophages contribute to angiogenesis-dependent diseases. *Eur J Cancer* **32**A: 2430–2437

Ribatti D, Nico B, Vacca A, Marzullo A, Calvi N, Roncali L and Dammacco F (1998) Do mast cells help to induce angiogenesis in B-cell non-Hodgkin's lymphomas? *Br J Cancer* **77**: 1300–1306

Risau W (1997) Mechanisms of angiogenesis. *Nature* **386**: 671–674

Rollins BJ (1997) Chemokines. *Blood* **90**: 909–928

Sheid B (1992) Angiogenic effects of macrophages isolated from ascitic fluid aspirated from women with advanced ovarian cancer. *Cancer Lett* **62**: 153–158

Sueishi K, Yasunaga C, Castellanos E, Kumamoto M and Tanaka K (1990) Sustained arterial injury and progression of atherosclerosis *Ann N Y Acad Sci* **598**: 223–231

Sunderkötter C, Steinbrink K, Goebeler M, Bhardwaj R and Sorg C (1994) Macrophages and angiogenesis. *J Leuk Biol* **55**: 410–422

Takahashi Y, Bucana CD, Lin W, Yoneda J, Kitadai Y, Cleary KR and Ellis LM (1996) Platelet-derived endothelial cell growth factor in human colon cancer angiogenesis: role of infiltrating cells. *J Natl Cancer Inst* **88**: 1146–1151

The Non-Hodgkin's Lymphoma Pathologic Classification Project (1982) National Cancer Institute sponsored study of classification of non-Hodgkin's lymphomas: summary and description of a working formulation for clinical usage. *Cancer* **49**: 2112–2135

Vacca A, Ribatti D, Roncali L, Ranieri G, Serio G, Silvestris F and Dammacco F (1994) Bone marrow angiogenesis and progression in multiple myeloma. *Br J Haematol* **87**: 503–506

Vacca A, Ribatti D, Fanelli M, Costantino F, Nico B, Di Stefano R, Serio G and Dammacco F (1996) Expression of tenascin is related to histologic malignancy and angiogenesis in B-cell non-Hodgkin's lymphomas. *Leuk Lymphoma* **22**: 473–481

Vacca A, Moretti S, Ribatti Pellegrino A, Pimpinelli N, Bianchi B, Bonifazi E, Ria R, Serio G and Dammacco F (1997) Progression of mycosis fungoides is associated with changes in angiogenesis and expression of the matrix metalloproteinases-2 and -9. *Eur J Cancer* **33**: 1685–1692

Vacca A, Ribatti D, Iurlaro M, Albini A, Minischetti M, Bussolino F, Pellegrino A, Ria R, Rusnati M, Presta M, Vincenti V, Persico MG and Dammacco F (1998) Human lymphoblastoid cells produce extracellular matrix-degrading enzymes and induce endothelial cell proliferation, migration, morphogenesis, and angiogenesis. *Int J Clin Lab Res* **28**: 55–68

Watanabe K, McCormick KL, Volker K, Ortaldo JR, Wiggington JR, Brunda MJ, Wiltrout RH and Fogler WE (1997) Regulation of host-mediated anti-tumor mechanism of cytokines. Direct and indirect effects on leukocyte recruitment and angiogenesis. *Am J Pathol* **150**: 1869–1880

Wolfe JE (1989) Angiogenesis in normal and psoriatic skin. *Lab Invest* **61**: 139–142

Xu Y, Hagege J, Doublet JD, Callard P, Sraer JD, Ronne E and Rondeau E (1997) Endothelial and macrophage upregulation of urokinase receptor expression in human renal cell carcinoma. *Hum Pathol* **28**: 206–213

© Cancer Research Campaign 1999
